# The gut microbiota pathway mechanisms of diabetes

**DOI:** 10.1186/s13568-023-01520-3

**Published:** 2023-02-08

**Authors:** Ousman Bajinka, Yurong Tan, Alansana Darboe, Isabella Gloria Ighaede-Edwards, Khalid A. Abdelhalim

**Affiliations:** 1 Department of Medical Microbiology, Xiangya School of Medicine, Central South University, Changsha, 410078 Hunan China; 2 China-Africa Research Center of Infectious Diseases, School of Basic Medical Sciences, Central South University, Changsha, 410078 Hunan China; 3 Vaccine & Immunity Theme, Infant Immunology, Medical Research Council Unit The Gambia at London School of Hygiene & Tropical Medicine (MRCG@LSHTM), Fajara, Gambia; 4 Department of Maternal and Child Health, Xiangya School of Public Health, Central South University, Changsha, Hunan China; 5 Izmir Biomedicine and Genome Center, Izmir, Turkey

**Keywords:** Diabetes, Gut microbiota, Inconsistencies, Gut-virome alterations, Gut-bacteriome–gut-virome-alterations

## Abstract

The contribution of dysbiotic gut microbiota configuration is essential when making reference to the metabolic disorders by increasing energy. It is important to understand that the gut microbiota induced metabolic disease mechanisms and inflammations. Thus it is imperative to have an insight into the state of all chronic subclinical inflammations influencing disease outcomes. However, from the emerging studies, there still exist inconsistencies in the findings of such studies. While making the best out of the reasons for inconsistencies of the findings, this review is designed to make a clear spell out as to the inconsistence of gut microbiota with respect to diabetes. It considered gut-virome alterations and diabetes and gut-bacteriome-gut-virome-alterations and diabetes as confounding factors. The review further explained some study design strategies that will spontaneously eliminate any potential confounding factors to lead to a more evidence based diabetic-gut microbiota medicine. Lipopolysaccharide (LPS) pro-inflammatory, metabolic endotoxemia and diet/gut microbiota insulin-resistance and low-grade systemic inflammation induced by gut microbiota can trigger pro-inflammatory cytokines in insulin-resistance, consequently, leading to the diabetic condition. While diet influences the gut microbiota, the consequences are mainly the constant high levels of pro-inflammatory cytokines in the circulatory system. Of recent, dietary natural products have been shown to be anti-diabetic. The effects of resveratrol on the gut showed an improved lipid profile, anti-inflammatory properties and ameliorated the endotoxemia, tight junction and glucose intolerance.

## Introduction

The functional medicinal approaches started with the experiment that involves replication of obese phenotypes of human discordant twin donors to mice through fecal microbiota transplantation (FMT) (Vrieze et al. 2012). This experimental phenomenon highlighted the need for microbiologists, gastroenterologists and clinicians to expand their scope in biomedical research. In a bid to elucidate the causative or mediatory role of the gut microbiota in diet-induced metabolic diseases, metabolic mediators such as gut microbiota are worth exploring (Vrieze et al. 2012; Yang et al. 2021a, b). Current evidence suggested a correlation or association between diabetes and the gut microbiota. The anti-diabetic activities exhibited by bioactive compounds and natural products that modulates the intestinal microbiota can have a significant effect on the diabetes disease mechanisms according to the emerging literature (Bajinka et al. 2020a, b; Gravitz 2012). A number of animal model studies have been conducted. However, no two animal species have shown uniformity in metadata of both the alpha and beta diversities occurring in the gut microbiota (Qin et al. 2012). Alpha diversity is the composition to determine ecological parameters within the groups while beta diversity gives the level of saturation for any given cohort per microbiome between groups.

The combination of the role of gut microbiota, the influence of natural products and their bioactive from the emerging evidence, is still not enough for clinical use. For instance the maturity-onset of diabetes of the young are as a result of single gene mutation, maternally inherited diabetes with deafness (MIDD) and mitochondrial mutations (Qin et al. 2012). With the robust molecular approaches in medicine and research, platforms like next-generation sequencing have laid foundations for microbiome related studies. This enables microbial surveys on a large-scale, which is based on informative marker genes. These may include; metagenomics for community gene inventories, 16S ribosomal RNA and metatranscriptomics for functional analysis (Teles et al. 2021).

Among overweight and obese individuals, Type 2 diabetes mellitus (T2DM) is one of the chronic metabolic diseases with a high prevalence worldwide. One out of every 3 adult Americans may develop diabetes by 2050 (Schmidt et al. 2021). Worldwide, around 463 million adults are confirmed to be living with diabetes mellitus in 2019 and upto 95% of are T2DM (Ballan and Saad 2021). The mechanism of T2DM is related to insulin resistance and this could be both genetic and acquired. With the advancement in medical research, the set of microbiomes that lead to insulin resistance and inflammation is established (Dash and Al Bataineh 2021). Diet and gut microbiota influence human physiology, which includes metabolism (Mokkala et al. 2021). In developing T2DM, normoglycemic individuals are found to develop a diabetes-like microbiota (Wang et al. 2021a, b). Gut microbiota is studied to modulate glucose metabolism through first-line pharmacotherapy using metformin to treat T2DM. In addition, it is found to strengthen intestinal permeability against lipopolysaccharides, increase in short-chain fatty acids, interaction with bile acids and consequently modulating the immune response (Lee et al. 2021).

A systematic review confirmed butyrate producers to dominate the gut of a healthy individual while *Lactobacilli* with increased plasma glucose was found to dominate the gut of T2DM patients. However, butyrate producers are even much higher than those taking metformin (Umirah et al. 2021). In addition to the metabolomics profile, the gut virome in an aggravate obesity-T2DM is also influenced by the external factors, which may include the geography and dietary lifestyle (Yang et al. 2021a, b). The functional output of the gut microbiota and microbial metabolites are promising in management of T2DM. The most recent findings with regards to impairing insulin signaling are but not limited to; lipopolysaccharides, imidazole propionate, trimethylamine while tryptophan metabolites and secondary bile acids may improve insulin signaling (Dovi, et al. 2022). Metagenomics operon has proven success with type 2 diabetes and this has laid foundations to therapeutic metagenomics and gene regulations (Zaidi et al. 2021). In expounding the association between the development of T1D and early life virome, there is a number of experimental evidence that confirmed epidemiological data. Metagenomic next-generation sequencing (mNGS) showed an increased association of fungi, bacteria and virus in association with T2DM (Huang et al. 2021). With mNGS, a meta-analysis indicated associations between T1D enteroviruses but this required more frequent sampling and follow-up studies (Faulkner et al. 2021). For pregnant women with T1D in the third trimester, lipopolysaccharides producing bacteria are enhanced against short-chain fatty acids producing bacteria (Roth-Schulze et al. 2021). T2DM patients have an imbalance Th17: Treg ratio, which is correlated with their gut microbiota dysbiosis (Essigmann et al. 2021). A stable coronary artery disease (SCAD) -type 2 diabetes (SCAD + T2DM) patients have been seen with an increased carbohydrates and aromatic amino acids. This is predictive of an enriched biosynthetic potential of the gut microbiome. Nitrogen metabolism potential is found to be increased by metformin against gut dysbiosis (Tian et al. 2021).

It is obvious that with a multi-omics research approach, the in-depth understanding of the global burden disease induced by T2DM will be thoroughly diagnosed and effective treatment options will be available (Wang et al. 2021a, b). Thus, this review will focus on the role of gut microbiota on T2DM based on the epidemiological clinical trials and some experimental evidence arising from animal model studies. Since this review is linking the gut microbiota with the forms of diabetes, other rare forms of diabetes such as maturity-onset of diabetes of the young will not be dealt with in detail. Although there is an enriched literature as to the gut power, the ability for gut microbiota to reverse the metabolic disorders and a health GI system, no extensive review is made on spelling out the inconsistency of gut microbiota with respect to diabetes. Thus this review hopes to make a clear cut between the several studies that give inconsistencies to gut microbiota in reversing diabetes' complications. It will consider gut- virome alterations and diabetes and gut bacteriome-gut-virome-alterations and diabetes as confounding factors. The review further hopes to explain study design strategies that will enable spontaneous elimination of any potential confounding factors that might influence the achieving of more evidence based diabetic-gut microbiota medicine.

## Pathways of interactions between gut microbiota and T2DM

**Fig. 1:**
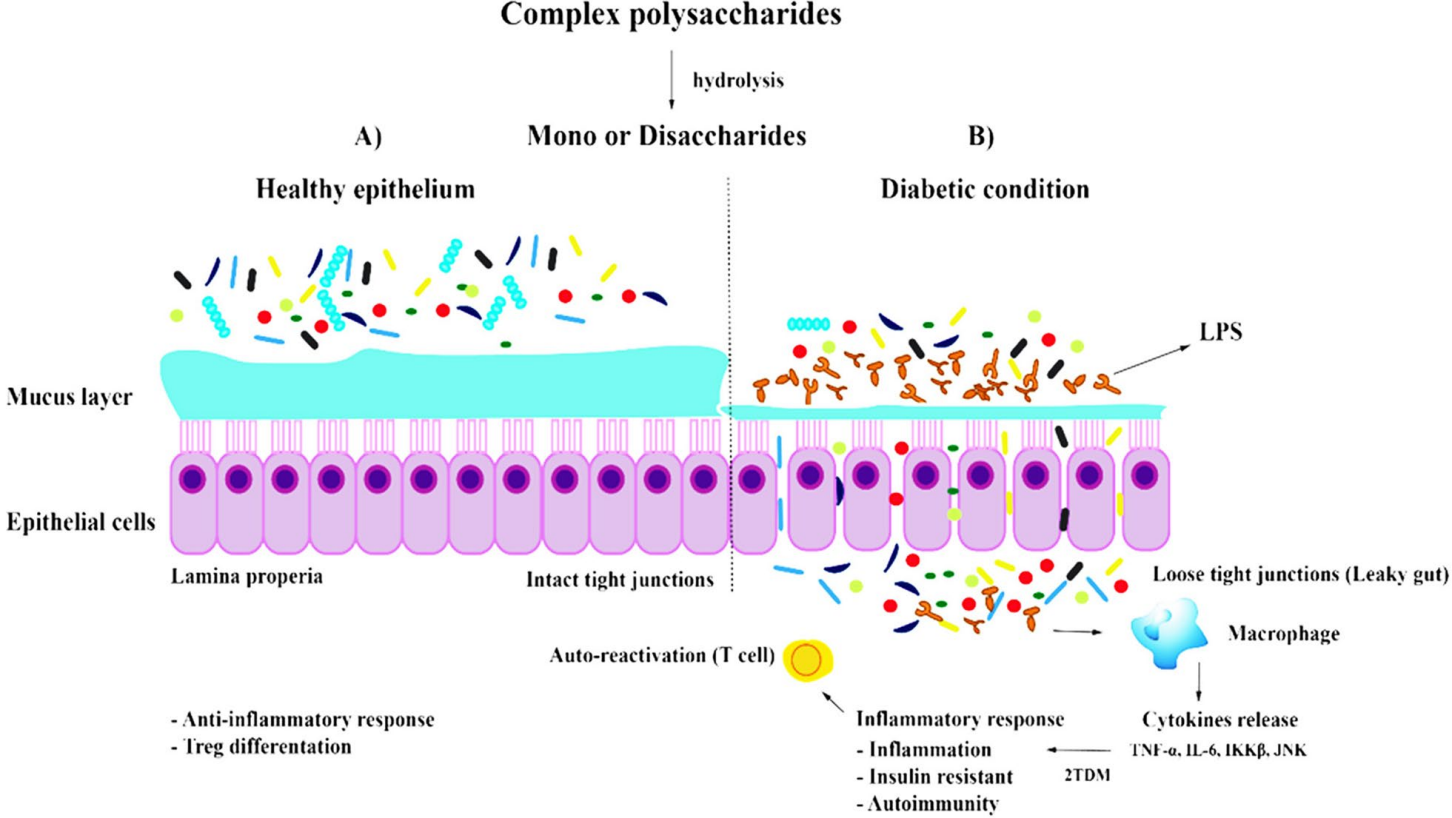
Intestinal flora interrogating the mechanisms and pathways of Type 2 Diabetes Mellitus

Mechanisms of gut microbiota in development of T2DM and its complications are explained in Fig. 1. The imbalanced intestinal microbiota of individuals with T2DM is attributed to factors such as medication including the excessive use of antibiotics, method of delivery (cesarean section), infant feeding and the diet of adult individuals. Genetic factors and diseases contributed but not as pronounced as the former. Stress and anxiety are also shown to affect intestinal dysbiosis (Li et al. 2019). Once this imbalance gut flora are established, it creates rooms for pathogenic gram negative bacteria to release LPS in the gut and this will lead to several chains of events. When LPS is secreted by a number of successful gram negative bacteria from the dysbiotic gut microbiota, it will be translocated to gut tissues. In these tissues, it will elicit the pro-inflammatory cytokine responses and series of pathways including triggering possible induction of insulin resistance before eventually leading to T2DM. LPS induces subclinical inflammation that leads to resistance for insulin and expansion of adipose tissue thereby disrupting the integrity of epithelial tight junctions. Once the mucosal epithelial barrier is impaired, followed by bacterial translocation, which is driven by the imbalance of the gut microbiota. It will cause elevated circulating and tissue microbe-associated molecular patterns (MAMPs) such as LPS and peptidoglycan (PG) (Luck et al. 2015). LPS found in gram negative bacteria activates Toll-like receptor 4 (TLR4) receptor on immune cells. Once released in the intestinal mucosal, it causes the release of cytokines such as TNF-α and IL-6 (Luck et al. 2015). TLR4 is expressed on macrophages and recognizes pathogen-associated molecular pattern (PAMP) on most pathogens. A reduction of SCFAs (acetate, propionate and butyrate) and secondary bile acids was observed in microbiota feed with high-fat diet. Note that bile acids are activators for glucagon-like peptide-1 (GLP1) secretion. GLP1 protects the body against insulin resistance (Luck et al. 2015). During the binding of LPS and TLR4, an extensive cell signaling pathway is activated. Thereafter, the inflammatory response, cytokine expression and secretion are induced. This marks the onset of permeability of intestinal tight junction (TJ). Reduction in the expression among proteins that compose tight junctions, such as claudin, zonula occludens-1 (ZO-1) and occludin lead to this permeability of the TJ. Note that TJ forms the epithelial barrier that helps to impede bacteria and other toxin-like particles from passing through intestinal lumen and reaching the circulation (Cruz-Morales et al. 2019).

The signaling pathway through the above mechanism is due to inflammatory responses triggered when LPS binds and activates TLR4 on innate cells such as macrophages. This activation dimerizes and recruits downstream adaptor molecules such as myeloid differentiation protein 88 (MyD88)/MyD88 adapter-like protein (MAL). MAL will then activate IL-1 receptor associated kinase (IRAK), TNF receptor-associated factor (TRAF6), and transform growth factors such as B-associated kinase 1 (TAK1), JNK and IKK complexes. Specifically, IKK complex will converge NF-_k_B, and this will be maintained in the inactive state. This status is enhanced by the nuclear factor of kappa light polypeptide gene enhancer in B-cells inhibitor (IB). Once NF-_k_B is translocated into the nucleus by degradation induced proteasomes, it activates the inflammatory cytokine responses. For an early factor in the establishment of insulin resistance, JNK and IKK must be activated by insulin receptor substrate (IRSs) serine phosphorylation (Bajinka et al. 2020a, b).

In metabolic endotoxemia, the administration of anti-inflammatory agents, 5-aminosalicylic acid have been proven efficacious in restoring the gut barrier. It reduces tissue microbiota dysbiosis, thereby inhibiting the onset of inflammation in some metabolic syndrome (Luck et al. 2015). It has been observed that the cross talk mediated by the gut microbiota can be abrogated with antibiotic intervention (Luck et al. 2015). The disrupting mechanisms leading to endotoxemia-induced phenotypes have been studied alongside the reduced inflammation markers. These are known for reverting intestinal permeability (Liu et al. 2020; Cani et al. 2008). Diet and gut microbiota can cause insulin resistance through low-grade systemic inflammation. This is characterized by constantly high levels of pro-inflammatory cytokines in the circulatory system such as TNF-α, IL-6, β kinase inhibitor (IKKβ), and c-Jun N-terminal kinase (JNK). All these molecules can phosphorylate insulin receptor substrate (IRS) and turn them into serine, which exerts a negative effect on insulin signaling and in some cases could lead to insulin resistance (Bajinka et al. 2020a, b).

## Inconsistencies in gut microbiota and diabetes

One distinct phenomenon that jeopardizes our reliability of microbiome data for T2DM in addition to the complicated analysis is non-biological zeros. Despite the fact that imputation microbiome data-mbImpute- can recover the non-biological zeros that might likely bring in irregularities, gathering metadata of sample taxon phylogeny and covariates from similar samples is not easy to achieve (Jiang et al. 2021). Thus, involving human clinical trials as to ethanol extract of mulberry leaves (MLE) in ameliorating dysbiosis in diabetes (Liu et al. 2021). It is obvious that the tensions between gene- and species-level of association from gene-based and indicators of cross-disease microbiome has still not gathered enough evidence based medicine. For instance, all liver cirrhosis, inflammatory bowel diseases and coronary artery disease except T2DM are associated with Streptococcus genus. However, exploring the gene-based on these positively associated, not all conformed to the correlations with Streptococcus genus (Tierney et al. 2021). In addition to viral and bacterial OTU that is yet to reach consistency as biomarkers of stages of T2DM is the insufficient data on molds. One instance is the filamentous molds in the order Mucorales causing mucormycosis as seen in poorly controlled diabetes mellitus patients (Sun et al. 2021).

## Gut virome alterations and diabetes

One study using shotgun metagenomic sequencing revealed the gut virome alterations in obese individuals with T2DM (ObT2). By comparing the bacteriome, virome and viral-bacterial correlations, the ObT2 were seen with a complete gut viral diversity and richness. Furthermore, intensive transkingdom correlations that occur between viruses and bacteria in lean controls and ObT2 were found to be significantly decreased. The common virus include; the phages of *Escherichia, Lactobacillus* and *Geobacillus.* Apparently, this study revealed that while gut virome may play a crucial role in development of T2DM, obesity with T2DM aggravates the obesity-associated virus signatures (Yang et al. 2021a, b). In the gut of children, the alterations of the virome are linked to type 1 diabetes in addition to malnutrition, celiac, diarrhea and inflammatory bowel disease (Fulci et al. 2021). However, the fecal virome of children of African and Asian countries with T1D could not show any consistency between these groups and the distant non-European population included in the study (Cinek et al. 2021).

## Gut bacteriome alterations and diabetes

From Vietnamese patients with T2DM, the gut microbiota reveals Firmicutes, Bacteroidetes and Proteobacteria. A significant reduction was detected through phylum Firmicutes and class *Clostridia* among ObT2 against the control group (Hoang et al. 2020). A reduced *Akkermansia muciniphila* and even further reduction are observed with obese individuals with ObT2. This reduction is positively correlated to insulin secretion with fibroblast growth factor concentrations (Zhang et al. 2021a, b). Phylum Firmicutes is abundant in the microbiota of saliva of Japanese patients with T2DM (Omori et al. 2022). The Firmicutes/Bacteroidetes (F/B) ratio is associated with a normal intestinal homeostasis and increased ratio of this is observed with obesity while decreased ratio are seen with inflammatory bowel disease (IBD) (Stojanov et al. 2020), and it is not a good news as per *Clostridia*, which is a causative bacteria of potentially deadly diseases like botulism and tetanus (Cruz-Morales et al. 2019). Moreover, the phylum Firmicutes and class *Clostridia* are found to be widely positively correlating with pro-inflammatory IFN-γ and negatively correlated with IL-6 (Umirah et al. 2021).

The metagenome analysis of old Chinese diabetic with frailty showed a reduced alpha diversity and *Collinsella* and *Butyricimonas* are common. Correlating these functional characteristics, an upregulated Epstein-Barr virus infection, biosynthesis of type II polyketide products, histidine metabolism and sulfur metabolism were observed against the down-regulated phenylalanine and butanoate (Peng et al. 2021). A reduced abundance of *Veillonellaceae* and*, Streptococcaceae*/*Pasteurellaceae* whereas an enhanced *Leptotrichiaceae* and *Neisseriaceae* was seen from a study (Balmasova et al. 2021). For gestational diabetes mellitus (GDM), specific taxonomic classes based on a systematic study revealed Proteobacteria, Bacteroidetes, Actinobacteria and Firmicutes to be positively related while *Bifidobacterium* and *Faecalibacterium* (butyrate producing) are negatively associated with GDM. The negative correlations mean glucose intolerance, inflammation and adiposity GDM women (Chu et al. 2021). Another inconsistency could be the close similarity between gout metagenomes to autoimmune instead of metabolic diseases like T2D. Despite systemic inflammation and urate degradation as presumptive non-invasive diagnostic markers induced by dysbiosis, T2D-like dysbiosis is not similar to arthritis diseases like gout (Kunasegaran et al. 2021). The studies on microbiome of diabetic foot ulcers (DFU) do not seem to produce great evidence based medicine (Schmidt 2021). However, a complex polymicrobial infection is suggested to be due to greater diversity and differential gene expression of enriched multispecies virulence, which includes; *Staphylococcus aureus* and *Streptococcus genera* in DFIs (Radzieta et al. 2021). To sum it all, the studies on bacterial characterization will see conflicting correlations among genera due to a number of reasons. Among these could be the inadequate lipid profile checking prior to study selections, the poorly inflammatory markers determination, imperfect monitoring dietary intake and deviations in the anthropometric measurements used to standardize grouping of study models (Que et al. 2021).

## Prospective study designs

Beta diversity instead of alpha diversity could be seen with significant differences and with an operational taxonomic unit (OTUs), an idea with probiotics would confer diabetes prevention and improvement (Umirah et al. 2021). *Lactobacillus plantarum*-pMG36e-GLP-1, an engineered strain is a promising diabetes treatment with its positive effects on faecal metabolomic profile and gut microbial composition (Luo et al. 2021).Chickpea extract as a natural Uyghur medicine in China has prebiotic effects as to the microbiota and metabolomics output and could be used in preventing diabetes (Li et al. 2021). Cholecystectomy, a surgical removal of the gallbladder intervention was found to alleviate long-term diabetes induced dysbiosis (Wei et al. 2021). Moreover, bariatric surgery could give detailed accounts on microbiota and how their metabolites can affect transcription in clinical outcomes of diabetes patients (Van Olden et al. 2021). Maternal cecal microbiota transfer (CMT) was also found to confer some form of protection against tissue-specific T1D injury (Zhang et al. 2021a, b). In this era of high-tech methods of molecular biology, establishing a system of biomarkers, clinical laboratory diagnostics and diagnostic research is very important. Families Prevotellaceae and Spirochaetaceae and in its composition are associated with chronic periodontitis with T2DM. Moreover, among the diagnostic values in assessing T2DM through microbiome, metabolic processes such as cysteine and methionine are increased against the decreased methane, sphingolipids, pyrimidine and the synthesis of fatty acids (Balmasova et al. 2021). FMT and lifestyle intervention (LSI) has been observed with positive correlations with liver stiffness and with reduced total and low-density lipoprotein cholesterol (Ng et al. 2022).

Since these studies with evidence based medicine as to gut microbiota and GDM are up to date, it is high time for clinicians to incorporate personalized medicine targeting microbiota modulation. This can be done through dietary intervention (Kunasegaran et al. 2021). However, western diets are metabolically less stable and when not monitored, might lead to complications like diabetes and obesity (Higgins et al. 2021). End-stage renal disease (ESRD), which is mostly caused by diabetic nephropathy (DN) can be prevented through the intakes of long chain omega-3 polyunsaturated fatty acids (dietary LCω3FA) (Perazza et al. 2021). Synbiotic based on an adlay seed extrusion cooked (ASEC)-based was found to ameliorate dysbiosis and consequently metabolic disorders (Chiou et al. 2021). Moreover, a combination of prebiotic and biotin supplementation has been found to prevent deterioration of metabolic conditions by limiting glycaemic deterioration (Belda et al. 2022). A bacterial taxa Ruminococcus torques as a keystone species for the degradation of resistant starch in gut microbiome (Ze et al. 2012) and also a predictive cardiac survival outcomes require more study to establish the type of OTU (Tian et al. 2021). Taking Actinobacteria as a biomarker of multidrug-resistant tuberculosis (MDR-TB) might be confounded based on the fact that this taxa is occurring even in patients taking anti-TB drugs (Wiqoyah et al. 2021). The damages done by free radicals and oxidative stress as determining factors for diabetes require a more robust study in restoring the antioxidant status in diabetic patients (Dvoretskaya et al. 2021). For impaired bacterial carbohydrate metabolism, robust interventional and longitudinal studies are required to elucidate on both periodontitis and with low grade inflammation induced by T2DM (Belstrøm et al. 2021). Considering the metabolic markers when involving gut microbiome, both female and male sex should be part of the model as in case of sex induced differences in metformin study (Silamiķele et al. 2021). Confounding factors like other diseases should be ruled out when associating gut microbiome with diabetes. For instance, a diabetic patient was found with pneumonia and right-sided parapneumonic effusion and following empirical antibiotics, *Prevotella pleuritidis* was identified (Galliguez et al. 2021).

## Natural dietary products as an anti-diabetes treatment

The availability of meta-analysis to investigate the role of gut microbiota in T2DM is not far fetched. Unlike T1DM, T2DM and its related metabolic disorders are known in mice to be diet-driven. Hyperglycemia, pancreatic β-cell decompensation and insulin resistance are the hallmarks associated with the clinical metabolic disorders of T2DM (Mayor et al. 2015). Factors triggering these conditions are the root causes of these and other forms of metabolic disorders (Luck et al. 2015; Lyssenko et al. 2008). According to the recent findings, both T2DM and obesity can increase the incidence of non-alcoholic steatohepatitis and other forms of cardiovascular disease (Lin et al. 2014). An instance is observed in the imbalance of microbiota seen among people with diabetes mellitus influence on the energy extraction of ingested food, intestinal permeability, transit time, systemic inflammation and mucosal immunity (Turnbaugh et al. 2009).

Modeling the gut microbiota with the intervention of Probiotics is proven efficacy. *Probiotics are live* microorganisms*,* when administered in adequate amounts; confer health benefits to the host (Gravitz 2012). It was shown that, *Bifidobacteria* spp and *Akkermansia* spp confer protective effects to gut microbiota (Lyssenko et al. 2008). The efficacies on the adherence and translocation of mucosal bacteria, tissue inflammation and insulin resistance are proven clinical relevance (Amar et al. 2011; Shin et al. 2014). One study, which incorporated the genera *Lactobacillus* spp and *Bifidobacterium* spp as probiotics, established a positive influence on the metabolic control of T2DM (Bajinka et al. 2023; Amar et al. 2011). Thus, there is still a need for further innovative investigation to ascertain if these interventions can control glycemic effects even though a number of studies produce correlating results as to some metabolic changes such as inflammation, oxidative stress and integrins (Anhê et al. 2015). Furthermore, the probiotics supplementation of polyphenols confers protective effects by increasing the population of *Akkermansia* spp. in diet-induced metabolic syndrome in mice (Cavallari et al. 2017). Nonetheless, a postbiotic (soluble metabolic byproducts) intervention of bacterial muramyl dipeptide was shown to reduce inflammation. This promotes insulin signaling in conditions of metabolic endotoxemia, glycemia and even in obesity via a pathway that involves NOD2 (Cao et al. 2019).

Natural dietary products and their bioactive compounds have proven their effectiveness in alleviating some conditions like diabetes hepatitis, cardiovascular, obesity and cancer (Shang et al. 2019; Luck et al. 2015). Available at a lower cost, natural products have been shown to be effective adjuvants in the therapy of T2DM (Qiu et al. 2020, 2021; Xu et al. 2015). The effects of natural products on T2DM is mainly described through the influence of gut microbiota, leading to the registration of a number of clinical trials in recent years. Gegen Qinlian decoction was given at moderate and high doses in a clinical trial. The treatment that is a form of Chinese herbal formula can significantly reduce the mean alterations in adjusted fasting blood glucose [FBG] and glycated hemoglobin A1C (HbA1c) levels. It does this by enriching the good bacterial composition with another benign species such as *Faecalibacterium prausnitzii* (Li et al. 2019, Singh et al. 2011). Another clinical trial involving 100 patients with T2DM reported that the disease condition can be ameliorated by Chinese herbal formula. This has been shown improvements in the glucose and lipid homeostasis by increasing the abundance of *Faecalibacterium *spp. (Singh et al. 2011).

High-fat diet decreases SCFAs, which are crucial in maintaining the integrity of tight-junction protein expression. This diet profoundly modulates microbiota and causes alteration that signals the onset of impairment of intestinal barrier. In order to restore eubiosis, diets rich in fibre change the expression of SCFAs. Extrathymic generation of anti-inflammatory regulatory T cells (Treg) is induced by expression of butyrate produced. The differentiation and IL-10 production is modulated by propionate. Insulin resistance is improved via reduction of macrophage infiltration in adipose tissue by Treg cells (Bajinka et al. 2020a, b).

One recent study showed that a modified gut microbial composition reduces the proportion of *Firmicutes* and the *Firmicutes-*to*-Bacteroidetes* ratio when resveratrol (RES) was administered. Some gut protective effects such as anti-inflammatory properties improvement, the lipid profile, ameliorated endotoxemia, intestinal barrier defects and glucose intolerance were observed (Chen et al. 2020). This study was conducted using mice fed a high fat diet and the finding opens doors forRES investigation as it may be useful for the treatment of insulin resistance and related metabolic diseases.

Metformin has an effect on entero-endocrine hormone, bile acid metabolism and gut microbiome alteration in patients with T2DM. Due to its complex effects; lowering glucose, which is attributed to the activation of AMPK and antagonism of glucagon mediated elevation of cAMP in hepatocytes, a possible novel therapeutic for T2DM is ensured. The therapeutic effects of metformin are relative to the mode of administering on patients. Intravenously does not improve glucose metabolism. In contrast, orally taken of metformin archived relevant therapeutically blood concentrations. The prolonged pharmacokinetics within GI tract that metformin exhibits due to the small polyvalent cation at physiological pH is achieved via oral administration (Napolitano et al. 2014).

Another beverage that is studied to reduce the postprandial glycemic response of bread is pomegranate polyphenols. This was not achievable when given as supplement and the extract. While some studies proposed insufficient mixing in the stomach and intestine could be the likely results, further study is required. Later in the postprandial period, microbial metabolites from pomegranate polyphenols modulate sugar metabolism. When consumed with a digestible carbohydrate such as bread, due to its polyphenols, it can reduce postprandial blood glucose spikes. The metabolic effects were seen in the reduction of the glucose IAUC by one-third (Kerimi et al. 2017). In order to ameliorate T2DM by regulating gut microbiota, natural products such as, insulin derivatives and medicine plants could enrich the gut microbiome with benign bacterial population. Beneficial bacteria and the most prominent ones being identified are *Faecalibacterium, Akkermansia muciniphila* and *Lachnospira species*. Despite the establishment of natural products and their bioactive properties as effective in modulating a normal gut microbiota, the mechanism of the metabolism ought to be investigated.

In conclusion, we recognised the power of gut microbiota and its related dysbiosis to form metabolic syndromes and disorders. The insulin resistance mechanism is active once passively depending on gut microbiota. Genetic modulation of the gut microbial metabolites and vaccine intervention are among the most recent strategies being developed in addition to anti-inflammatory agents for therapeutics of chronic metabolic diseases. Once the trialogue occurring among nutritional status, gut microbiota and immune system are elucidated, the number of breakthrough interventions will probably lead to a lifetime novel therapeutic opportunities for chronic metabolic diseases. Thus, the mechanistic roots of the ground breaking therapeutic strategies should be designed to target the host immune and nutrient-sensing pathways. This will enable us to synthesize novel innovative interventions that could lead to new treatments of chronic metabolic diseases. This review made emphasis on looking inwardly as to the conditions in which participants are recruited to take part in gut microbiota study. While the review based its argument on gut-virome alteration and gut-bacteria-gut-viral alterations induced confounding factors in studies focused on gut manipulating microbiota reversing diabetic complications, there is need to establish confounding-free state of art research design that will lead a consistent mega-studies and establish evidence based medicine. They recommended the study designs be directed so they can determine the association between diabetic patients’ biochemistry, haematology, metabolomics, metagenomics and proteomics to eliminate the possible confounding factors in the study.

## Data Availability

Not applicable.
